# The impact of new research technologies on our understanding of environmental causes of disease: the concept of clinical vulnerability

**DOI:** 10.1186/1476-069X-8-54

**Published:** 2009-11-30

**Authors:** Paolo Vineis, Aneire E Khan, Jelle Vlaanderen, Roel Vermeulen

**Affiliations:** 1 MRC/HPA Centre for Environment and Health, Imperial College, UK; 2 Institute for Risk Assessment Sciences, Utrecht University, Utrecht, the Netherlands

## Abstract

In spite of decades of epidemiological research, the etiology and causal patterns for many common diseases, such as breast and colon cancer or neurodegenerative diseases, are still largely unknown. Such chronic diseases are likely to have an environmental origin. However, "environmental" risks have been often elusive in epidemiological studies. This is a conundrum for current epidemiological research. On the other side, the relative contribution of genes to chronic diseases, as emerging from GWAS, seems to be modest (15-50% increase in disease risk). What is yet to be explored extensively is a model of disease based on long-term effects of low doses of environmental exposures, incorporating both genetic and acquired susceptibility ("clinical vulnerability"), and the cumulative effects of different exposures. Such a disease model would be compatible with the weak associations found by GWAS and the still elusive role of many (low-level) environmental exposures. We also propose that the introduction of "-omic" high-throughput technologies, such as transcriptomics, proteomics and metabolomics, may provide, in the next years, powerful tools to investigate early effects of environmental exposures and understand the etiology of common diseases better, according to the "clinical vulnerability model". The development of "-omics", in spite of current limitations and lack of sound validation, could greatly contribute to the elucidation of the disease model we propose.

## Introduction

We are still far from completely understanding the etiology and causal patterns for many common diseases, such as breast and colon cancer, or neurodegenerative diseases. In spite of decades of epidemiological research, the etiology of many such conditions is still largely unknown.

For a long time it has been hypothesized that most chronic diseases have an environmental origin (using the term "environment" in a broad sense, to indicate essentially what is not due to genetic predisposition). This claim was based on descriptive data showing the broad range of incidence rates in different parts of the world, the rapid temporal changes - such as those currently occurring in China and India - and the crucial observation of incidence rates in migrant populations. The latter unequivocally showed that migrants rapidly acquire - sometimes already in the first generation after migration - the risk of disease that is typical of the population where they move.

However, "environmental" risks have been largely elusive in epidemiological research, and there is still much debate on the real impact of the environment, whose role tends to be overshadowed in recent times by the successes of genetics, in particular Genome-wide association studies (GWAS). Clearly, we need a disease model that allows us to encompass both the mass of genetic data coming from GWAS and the still elusive role of many environmental exposures.

## Genes vs. environment

**Figure 1:**
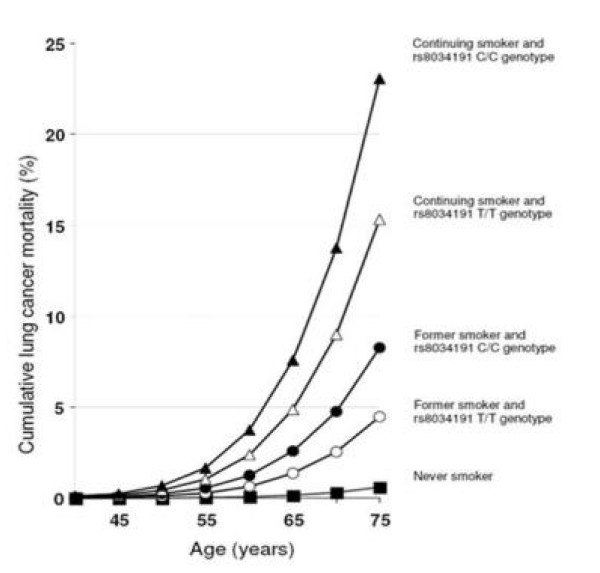
**Cumulative risk of lung cancer by rs8034191 genotype**. Relevance of smoking and of rs8034191 genotype to lung cancer mortality in men aged 45-75 years. Cumulative risk (in the absence of other causes of death) based on national lung cancer death rates for men in Poland in the year 2000, assuming that the prevalence of current smoking, former smoking, and never smoking are as in this study and that the relative risks for lung cancer incidence and mortality are similar (Hung *et al *2008) [1].

The technological revolution in genetics, which in 2007-2009 led to the identification of a number of novel genes for several common diseases, is inspiring but at the same time a little disappointing. Most highly-penetrant genes had already been uncovered by linkage studies, so that "genome-wide scans" could mainly identify low-penetrant genes. It is a fact that - with a few exceptions - what has been found is genetic variants weakly associated with chronic diseases, with relative risks of 1.15-1.5. These risks are much lower than some known environmental exposures, and of the same order of magnitude of environmental exposures that are usually rejected as too weakly associated with disease to be meaningful or interpretable. For example, Hung et al have found an allelic relative risk of 1.3 for a gene variant associated with lung cancer, in the putative nicotinic acetylcholine receptor subunit on chromosome 15q25 [1]. The genotype nicely combines with the habit of smoking, increasing the probability of developing lung cancer in smokers (Figure 1) [1]. However, it is clear from the figure that the effect of smoking is by far greater than the effect of the genotype. The former increases the cumulative risk in life from less than 1% (non smokers who have never smoked) to about 15-20% (current smokers), while the effect of the genotype is a small additional amount (~1 to 2%) that does not seem to contribute much to the Public Health problem of lung cancer.

Of course genetic research is not tailored for Public Health purposes. It can be worthwhile, instead, (a) for the investigation of disease mechanisms, (b) to shed light on potential therapies, or (c) to suggest clues for unidentified etiological agents in the case of diseases whose etiology is still unknown. It is unclear, however, how goals (b) and (c) can be met if the relative contribution of genes is so small (15-50% increase in disease risk) being in the same order of magnitude of relative risks found for unclear and controversial environmental exposures, such as electromagnetic fields and leukemia, or some pesticides and prostate cancer. It is therefore useful to reflect on the potential reasons why weak genetic associations (generally) are perceived as much more noteworthy than environmental associations.

1. One reason - perhaps the main one - is certainly the greater robustness of technical detection of gene variants compared to environmental exposures. It is estimated that the error rate with common genotyping techniques is less than 10%, while it can be as high as 50% for environmental exposure assessment, at least for dichotomous exposure variables. Moreover genes are stable in the course of life, while environmental exposures are changing over time (let us think of diet) leading to more inherent measurement error in environmental exposure than in gene variance assessment.

2. A second obvious reason is replication. In the study mentioned above on lung cancer, the association was found in five independent studies. This usually is not the case for environmental exposures, where replication is not easily achievable. For example, a cohort study from Belgium suggested that high levels of cadmium in urine are associated rather strongly with lung cancer [2]. The study is strong (though small), because exposure assessment is based on a biomarker reflecting long-term exposure (i.e. with much higher accuracy than usually available in epidemiology). However, the study is not easily reproducible, because there are not many other populations with similar levels of environmental exposure to cadmium, and there might be several confounders or effect modifiers that either obscure the association with cadmium or change the strength of the association. This is commonly observed in environmental epidemiology, i.e. a number of small-medium size associations that are difficult to reproduce in different settings, both because of methodological difficulties and because of variable study contexts.

## Low doses and acquired susceptibility to disease

In spite of the limitations of environmental epidemiology, in particular of exposure assessment, we have seen recently increasing ability of epidemiological tools to consistently unravel the effects of low-level environmental exposures. The cases of air pollution and environmental tobacco smoke (also called ETS, secondhand smoke or passive smoke) are exemplar. In the seventies and eighties many researchers thought it was impossible to detect plausible causal associations with such low-level exposures. The underlying idea was that "noise" (bias, confounding) was larger than the signal. However, a large number of well-conducted studies have been published since: in the case of ETS more than 60 studies show (with few exceptions) increased risks of lung cancer in the order of 1.25, i.e. the same magnitude as the gene for the nicotine acetylcholine receptor [3]. For air pollution, an association with lung cancer has been reported in six cohort studies. One of these studies is sufficiently large as to show the association in non-smokers [3,4]. Again, the relative risk is around 1.25. In both cases biomarkers, such as cotinine or NNK for ETS and DNA adducts for air pollution, have contributed to make the association more plausibly causal.

We have thus to acknowledge that ETS and air pollution (mainly due to traffic exhaust) are able to induce, after long-term exposure, chronic diseases such as cancer and also coronary artery disease. The interesting observation is that this happens at dose levels that are much lower than those of tobacco smoke or "classical" carcinogens. For example, exposure to ETS occurs at levels that are 1/100 compared to active smoking [5].

**Figure 2:**
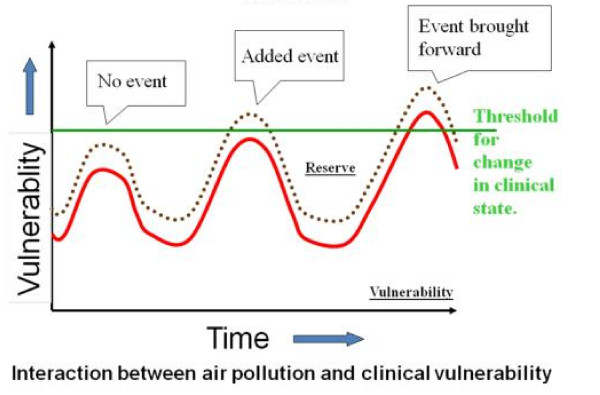
**Vulnerability plus exposure events change the physiological state, but a reserve is present**. When the reserve is overtaken, clinical manifestations appear. From http://www.sahsu.org/jubilee_presentations/Anderson.ppt#392,21, courtesy of R Anderson.

One potential explanation for the effects of low doses and the absence of a threshold is based on acquired susceptibility and the cumulative effects of different exposures. As defined by Rothman and Greenland, "the cause of a disease event is an antecedent event, condition or characteristic that was necessary (given that all other conditions are fixed) for the occurrence of the disease at the moment it occurred" [6]. Said in other words, a cause can also be viewed as something that "completes an incomplete causal chain" [7] or precipitates a chain of events, which creates a state of vulnerability. This concept of "clinical vulnerability" is expressed in Figure 2[8]. Exposure to low levels of e.g. ETS or air pollution is not a "cause" of cancer in itself (like an accident is the cause of a death), but because it occurs on top of pre-existing vulnerability. This could well explain why small changes in environmental exposures can have big effects, if they occur in a population of vulnerable subjects.

Vulnerability can be acquired or genetically-based. The concept of acquired "clinical vulnerability" is related to previous insults/pathophysiological changes that predispose to disease, as expressed in Figure 2. An example is the finding of a greater effect of ETS among ex-smokers compared to non smokers who have never smoked in a large prospective investigation [9]. It is plausible that ex-smokers have a greater vulnerability because of already existing mutations or epigenetic changes, so that further exposure to ETS leads to selection and clonal expansion of mutated cells.

Another type of vulnerability (more often called hypersusceptibility) is genetically determined. Many years ago we showed that subjects with the genetically based NAT2 slow acetylator genotype could have greater susceptibility to adverse biological and/or health effects by tobacco smoke related arylamines at lower levels of exposure rather than at higher levels [10]. Our reasoning was that on very rare occasions, e.g. among people exposed to high doses of potent carcinogens, the whole population or a vast majority develops cancer. This is what happened among British chemical workers exposed to 2-naphthylamine in the 1950s and before. For example, all 15 workers exposed to 2-naphthylamine in a plant developed bladder cancer, probably one of the few examples of a "sufficient" exposure in the history of carcinogenesis [11]. It is clear that in that case genetic susceptibility was totally irrelevant. In most cases, however, when exposure levels are lower or much lower, genetic susceptibility can play a role in modulating individual risk.

## The contribution of intermediate biomarkers and '-omics'

The concept of acquired "clinical vulnerability" is related to previous insults/pathophysiological changes that predispose to disease. Intermediate markers and specifically '-omics' could be particularly useful in tracing the "history" of such insults and in reflecting the cumulative effect of different exposures.

## Intermediate Biomarkers

Intermediate biomarkers directly or indirectly represent events on the continuum between exposure and disease. Intermediate biomarkers can provide important mechanistic insight into the pathogenesis of environmental diseases. As such, they complement classic epidemiological studies that use disease endpoints. Examples of early biologic effect biomarkers include measures of cellular toxicity, chromosomal alterations, DNA, RNA and protein expression, and early non-neoplastic alterations in cell function (e.g., altered DNA repair, altered immune function). For maximum utility, an intermediate biomarker must be shown to be predictive of developing disease. The criteria for validating intermediate biomarkers have been discussed by Schatzkin and colleagues [12,13] and focus on the calculation of the etiologic fraction of the intermediate endpoint, which varies from 0 to 1. The closer the etiologic fraction is to 1, the more the biologic marker reflects events, either directly or indirectly, on the causal pathway to disease. For instance, bone-marrow toxicity has been linked to future risk of leukemia. Therefore, environmental exposures that lead to bone-marrow toxicity are potentially leukemogens. Bone-marrow toxicity can be measured by studying peripheral blood cell counts including circulating progenitor cells. Studies based on such endpoints have the advantage that the biological marker can be measured quantitatively and therefore increase the power as compared to dichotomous (rare) disease endpoints. Furthermore, these studies can be carried out using cross-sectional or semi-longitudinal study designs in which exposure can be accurately assessed. An example of the use of intermediate markers is a recent study on hematological effects of benzene at low levels of exposure [14]. In this study peripheral blood cells and progenitor cell colony formation were significantly decreased among low exposed individuals in a dose-dependent matter. Moreover, these effects occurred at exposure levels that were lower than previously thought safe [14].

## -Omics

"-Omics" tools can be directly applied to samples from an epidemiologic case-control or cohort study to better characterise intermediate pathways, potentially providing the 'missing links' among exposures, genes, and diseases [15]. The term "-omics" generally refers to the rigorous study of a complete set of biological molecules with high-throughput techniques [16]. One of the main hallmarks is the departure from traditional molecular biology focusing on a single biological structure (e.g. a single gene or protein) to a more comprehensive analysis of biological systems.

Cells and tissues alter their metabolism or gene expression in response to exposure as indicated by, for example, the induction of specific groups of genes in response to heat shock, hypoxia, or osmotic stress [17-19]. These responses can produce patterns of specific changes in gene expression, proteins, or metabolic profiles that reflect exposure to a particular agent or class of agents which can be interpreted as a "history" of such exposures and might reflect the cumulative effect of different exposures. Some of the "-omic" technologies in environmental health that offer new opportunities to detect and quantify these changes are discussed below (also see table 1). We propose some examples of uses of "-omics" in environmental health, mainly to elucidate which kind of contributions we may expect.

**Table 1:** "-omics" in Environmental Health (except genomics).

	Transcriptomics	Epigenomics	Proteomics	Metabolomics
**Definition & sources of samples**	Focuses on gene expression profiling, which is the assessment of the mixture of mRNAs that is present in a specific biological sample. mRNA from all types of tissues can be used.	Focuses on epigenetic changes in DNA and chromatin and on their impact on regulation of gene expression. These changes are independent of DNA sequence, and are involved in 'gene silencing'. Genomic DNA from all types of tissues can be used.	The study of proteins carried out to understand the amount and functioning of proteins in biological systems. All types of tissues and biofluids can be used for proteomic analysis. Plasma, a readily accessible fluid is most commonly used.	The measurement of all the metabolites in a specified biological sample. Samples of a biological origin are analyzed using techniques that produce simultaneous detection, thereby providing metabolite profiles. It is mainly conducted on bio-fluids such as urine or plasma, and sometimes tissue extracts, *in vitro *cultures, and supernatants.
**Tools** **used**	A quantitative technique is used to associate differences in mRNA mixtures originating from different groups of individuals to phenotype differences between the groups. It is strongly constrained by the intrinsic heterogeneity and instability of mRNA. Both the presence of specific forms of mRNA and the levels in which these forms occur are parameters that provide information concerning gene expression. Microarray is the most commonly used research tool.	Currently limited to the analysis of gene methylation profiles. The standard method consists in sequencing of DNA segments containing potentially methylated fragments after modification of DNA with sodium bisulfite, which selectively modifies non-methylated cystosine, thus creating a base change which does not take place when cytosine is methylated. This change can be identified either by sequencing or by genotyping using custom-made 'genome-wide' oligonucleotide arrays.	Reasonable consensus on the use of mass spectrometry for final identification of proteins/peptides but technologies for sample fractionation are variable. These technologies rely on 3 main approaches: (1) 2 dimensional electrophoresis to select protein spots that are eluted from gels and analysed by MS; (2) combined chromatographic approaches to trap abundant proteins and separate the less abundant ones before MS; (3) use of matrixes of immobilized chemicals to adsorb proteins based on different criteria (charge, hydrophobicity, affinity, binding to specific ions), followed by desorption and MS (SELDI/TOF).	A variety of analytical metabolic profiling tools used include H NMR spectroscopy and MS with a prior online separation step like high-performance liquid chromatography, ultra-performance liquid chromatography, or gas chromatography.
**Analysis**	Hierarchical clustering and principal component analysis are commonly used statistical approaches for the identification of gene sets. For the interpretation of the relevance of differently expressed gene sets, data analysis approaches that are able to integrate microarray data with prior knowledge on the involvement of genes in biological processes are needed.	Conventional statistical methods are used to detect disease - gene methylation associations. Analyses of genome-wide methylation data involve hierarchical clustering and discriminant analysis.	Current approaches to analyze the protein composition of biofluids or tissue homogenates generate large amounts of data. A variety of statistical methods are currently available to epidemiologists including discriminant analysis. Both random (measurement) and systematic (bias) errors should be considered as a necessary component of proteomic analyses.	Data generated by these analytical techniques are often combined with multivariate data analysis, e.g. (orthogonal) partial least square, clustering, discriminant analyses and other similar approaches for generating and interpreting the metabolic profiles of the investigated samples.

## Transcriptomics

Transcriptomics is a mature technology, however still strongly constrained by the intrinsic heterogeneity and instability of mRNA. It focuses on gene expression profiling, which can be used to determine which genes are differently expressed as a result of changes in environmental conditions. A study typically involves a group of individuals with similar exposure level or phenotype (e.g. disease status) and compares the gene expression profile of this group to the profile of a reference group. To avoid the problems of large interindividual variations compared with the small changes caused by environmental exposure, Wang et al conducted a self-controlled study design, with measurements made before and after individual exposure to welding fumes. The group was stratified according to smoking status (which profoundly affected the expression profiles). Nonsmokers after exposure to welding fumes exhibited altered gene expression in 35 genes from eight functional pathways, including processes related to oxidative stress, proinflammatory responses, phosphate metabolism, cell proliferation, and apoptosis [20].

A study by Lu et al used a human cancer cDNA expression array to detect differential gene expression patterns determined in liver tissue samples from arsenic exposed individuals and healthy liver tissues [21]. Approximately 60 genes (10%) were differentially expressed in arsenic exposed human liver tissues compared to controls. The differentially expressed genes included those involved in cell-cycle regulation, apoptosis, DNA damage response and intermediate filaments. The observed gene alterations appeared to be reflective of hepatic degenerative lesions seen in arsenic-exposed patients.

However, transcriptomic changes are likely to be temporary, i.e. reflect short-term exposures, and unlikely to express the long-term changes that we need in order to investigate chronic diseases.

## Epigenomics

Epigenomics involves the study of epigenetic processes, which are independent of DNA sequence and are involved in the inhibition of gene expression (gene silencing), on a large scale [22-24]. It benefits from the robustness of technologies for genomics. Altered gene silencing plays a causal role in human diseases [25-28]. Epigenetic modifications may provide a plausible link between environmental exposures and alterations in gene expression that might lead to disease phenotypes. A large amount of laboratory animal studies already provide evidence that supports the role of environmental epigenetics in disease susceptibility [25]. Epigenomics is mainly based on a few comprehensively studied mechanisms: (a) DNA methylation, which takes place at the carbon-5 position of cytosine in CpG dinucleotides, (b) changes to the chromatin packaging of DNA by post-translational 'histone modification', and more recently (c) changes in RNA interference by non-coding RNAs such as microRNA and siRNA.

In mice, maternal dietary methyl-donor supplementation with folic acid, vitamin B, choline and betaine was shown to result in an increase in DNA methylation at CpG sites, thus demonstrating that the effect of a mother's diet during pregnancy on the adult phenotype of her offspring was directly linked to DNA-methylation changes in the epigenome [25]. A few more studies on maternal dietary exposure, e.g. to phytoestrogen genistein during gestation given at a level comparable to that of human consumption with high soy diets, are particularly interesting as these results suggest the possibility that hypermethylating dietary supplements could reduce the effects of environmental toxicants, which tend to cause DNA hypomethylation [25].

Recent experiments have shown that epigenetic changes are associated with chromatin remodelling and regulation of gene expression that underlie the development of the metabolic syndrome, and to other common diseases like atherosclerosis and type 2 diabetes [29]. Deciphering epigenetic processes should allow us to target the development of new diets and drugs to prevent aberrant gene silencing, which may be involved in resistance to treatment. The importance of DNA methylation in cancer has also been established, and research now includes the mechanisms by which other chromatin modifications play a role in cancer development [30]. In particular, the link between covalent histone modifications and nucleosomal remodeling is increasingly being explored [30,31].

Also several environmental exposures have been associated with altered levels of gene methylation. In one study, decreased repeated-element methylation in gene LINE-1 was observed after exposure to traffic particles (beta = -0.09; 95% CI, -0.17 to -0.01; P = 0.03) [32]. Also airborne benzene was associated with a significant reduction in LINE-1 (-2.33% for a 10-fold increase in airborne benzene levels; P = 0.009) and AluI (-1.00%; P = 0.027) methylation. Hypermethylation in p15 (+0.35%; P = 0.018) and hypomethylation in MAGE-1 (-0.49%; P = 0.049) were associated with increasing airborne benzene levels [33] In another study, Rusiecki et al found inverse correlations between global methylation levels and POP (Persistent Organic Pollutant) concentrations in the blood of Inuit populations [34]. Linear regressions, adjusting for age and cigarette smoking, showed statistically significant inverse linear relationships mainly for the Alu assay for p, p'-DDT (1,1,1-trichloro-2,2-bis(p-chlorophenyl)ethane; beta = -0.26), p, p'-DDE [1,1-dichloro-2,2-bis(p-chlorophenyl) ethylene; beta = -0.38], beta-hexachlorocyclohexane (beta = -0.48), oxychlordane (beta = -0.32), alpha-chlordane (beta = -0.75), mirex (beta = -0.27), sum of polychlorinated biphenyls (beta = -0.56), and sum of all POPs (beta = -0.48). Linear regressions for the LINE-1 assay showed beta estimates of similar magnitudes to those using the Alu assay, however, none was statistically significant.

## Proteomics

The proteome consists of all proteins present in specific cell types or tissues and, in contrast to the genome, it is highly dynamic over time, between cell types and in response to the environment. Proteomics is perhaps the least mature of the "-omic" technologies. Whether agent-specific changes can be identified is a key question to be answered about proteomic approaches (note, the same issue applies to other "-omics", such as metabolomics) and the issue will be resolved only after a much larger body of proteomic data on exposed cells, tissues, and biofluids is collected and analyzed [35]. Proteomic approaches are twofold: the first is the standardisation of high-throughput methods, and the second is to combine extensive protein fractionation, removal of abundant proteins and tandem MS/MS analysis of peptide sequence data to allow protein identification.

Joo et al performed proteomic analysis of plasma proteins among workers exposed to benzene and matched unexposed controls, and associated up-regulation of specific proteins with lymphocyte DNA damage [36]. Using MS and Western blot, they found statistically significant differences in protein profiles between the exposed and unexposed subjects. The specific proteins that were found to be up-regulated in benzene exposed workers were TCR beta, FKBP51 and MMP13. Vermeulen et al examined the impact of benzene on the human serum proteome in a study of benzene- exposed workers and unexposed controls, where protein-expression patterns were analysed by SELDI-TOF MS. They found that three proteins were consistently down-regulated in the exposed group compared to controls and all proteins were highly inversely correlated with individual estimates of benzene exposure (r > 0.75) [37].

Proteomics has been useful in the field of cancer, particularly in the potential discovery of new biomarkers that may improve the diagnosis of prostate and other cancers [38]. Proteomic approaches may also provide insights into the disease mechanism of rheumatic disease [39,40]. Lopez-Pedrera reviews how recent genomic and proteomic approaches have provided new insights into autoimmune diseases [41]. Obesity, which is caused by a combination of genetic predisposition and environmental factors, is an area where proteomic approaches are also being applied. Barcelo-Batllori and Gomis discuss the application of proteomics in understanding the pathogenesis or the molecular mechanism of obesity [42].

However proteomics is still open to several methodological problems that have not been solved yet. The most controversial example has been in reporting a distinctive pattern of low-molecular weight proteins that characterises the sera of women with early-stage ovarian cancer that is very rarely found in control sera [43]. The study was later refuted by others who offered alternative interpretations of the initial observations [44]. This was a case where the basic epidemiologic principle of treating samples from cases identically to those from controls was possibly not followed, with the apparent differences between cases and controls reflecting artefacts of sample handling [45].

## Metabolomics

Changes in gene and protein expression can alter metabolism in particular ways that can provide distinct signatures. The metabolome is the biochemical network consisting of small molecules (e.g. lipids, vitamins) also known as metabolites. Metabolites are involved in energy transmission in cells by interacting with other biological molecules following metabolic pathways. The metabolome is highly variable and time dependent and consists of a wide range of different chemical structures. The most important challenge in this field is to acquire qualitative and quantitative information on metabolites that occur under normal circumstances in order to be able to detect perturbations in the complement of metabolites due to changes in environmental factors. Global metabolomic profiling may be more amenable than transcriptomic or proteomic profiling to high-throughput screening, as suggested for example by studies showing that the main yeast metabolome consists of fewer than 600 low-molecular-weight compounds. It should be noted however that the yeast has only 4,000 genes and is a unicellular organism, representing thus an oversimplified model to study humans. Substantial metabolomic analysis of exposure and effects will be needed to address the possibilities offered by this approach [35,46]. Global metabolomic analysis in exposure studies in laboratory animals has just begun, but the low-level exposures of interest in large populations have not yet been addressed.

Metabolomic studies are performed with noninvasive samples such as biofluids and breath condensate as well as on tissues in vivo. Profiles within a tissue or cell are compared with profiles in biologic fluids or with cell secretion products to understand the metabolic consequences of xenobiotic-induced toxicity. Examples of this approach are the studies by Waters and colleagues, where NMR and pattern recognition analysis were used to investigate time-related metabolic effects of α-naphthylisothiocyanate on liver, urine, and plasma in laboratory animals [47,48]. Waters and colleagues found an association between hepatic lipidosis and increased urinary excretion of taurine and creatine. In addition, there was reduced urinary excretion of intermediates in the tricarboxylic acid cycle and increased excretion of plasma ketone bodies. These studies enabled a clearer understanding of key metabolic effects during the development of and recovery from toxic lesions. In the past, there was a failure in specificity for cancer, which hampered biomarker discovery by metabolomics, but technologies like NMR and MS are being reapplied to study a range of cancers [49,50]. Recently a metabolomic study has been successfully applied to epithelial ovarian cancer and was apparently able to discriminate women with epithelial ovarian cancer from healthy controls, based on 1H NMR spectroscopic profiles of preoperative serum specimens [49].

Holmes et al [51] used a large-scale exploratory analytical approach to investigate metabolic phenotype variation across and within four human populations, based on 1H NMR spectroscopy. Metabolites discriminating across populations were then linked to data for individuals on blood pressure. They analysed spectra from two 24-hour urine specimens for each of 4,630 participants from the INTERMAP epidemiological study, involving 17 population samples in China, Japan, UK and USA. They showed that urinary metabolite excretion patterns for East Asian and western population samples, with contrasting diets, diet-related major risk factors, and coronary heart disease/stroke rates, were significantly differentiated (P < 10(-16)), as were Chinese/Japanese metabolic phenotypes, and subgroups with differences in dietary vegetable/animal protein and blood pressure. Among discriminatory metabolites, they quantified four and showed associations (P < 0.05 to P < 0.0001) of mean 24-hour urinary formate excretion with blood pressure in multiple regression analyses. Mean 24-hour urinary excretion of alanine (direct) and hippurate (inverse), reflecting diet and gut microbial activities, were also associated with blood pressure [51].

## Discussion

The prevailing model of "environmental disease" for decades has been influenced by toxicology, with single agents evaluated at a time, and dose as a key issue. A more recent model, which was not overall very successful, was that of "gene-environment" interactions in which environmental agents at low doses interact with individual genetic susceptibility. What has not been explored extensively yet is another potential model for the effects of low doses and the absence of thresholds, represented by acquired susceptibility and the cumulative effects of different exposures. The concept of "clinical vulnerability" could explain why small changes in exposure can have big effects, if they occur in a population of vulnerable subjects. The concept of acquired "clinical vulnerability" is related to previous insults/pathophysiological changes that predispose to disease.

The new "-omics" can be extremely useful in tracing the "history" of such insults and to reflect the cumulative effect of different exposures. However, the success of "-omics" applications to epidemiology will depend on a coherent and effective model of disease, that addresses issues such the role of environmental low-level exposures.

The ultimate goal of using "-omics" technologies to identify environmental causes of disease is to derive an integrated view of the biological processes involved in the continuum from exposure to disease. Although high-sensitivity and data-rich toxicogenomic approaches may already be feasible for analyzing human responses to environmental stressors in some settings, applying these technologies to human populations exposed to low-level environmental contaminants will require considerably more development. In this context, the integration of information from multiple toxicogenomic approaches may provide significantly more analytical power than any one approach alone.

Current limitations of "-omics" include low reproducibility across laboratories, high intra-individual variability that hampers inter-individual comparisons, high costs, difficulties in data analysis and uncertainties in biological interpretation (see table 2). Type I, II, and III errors need to be addressed. Type I error refers to false positive results deriving from the large number of comparisons. This can be tackled with in-built replication with repeat measures in independent studies. Type II error derives from the relative lack of sensitivity of some techniques and the ensuing false negatives issue. Type III error refers to the lack of relevance of some biomarkers, with uncertain biological meaning, for the interpretation of exposure disease relationships. Lumbreras et al discuss some of the sources of errors in regard to "-omics" based diagnostic techniques and their solutions, and the effects on diagnostic accuracy [52,53].

**Table 2:** Using "-omics": advantages and limitations

Advantages	Use in large, hypothesis-free investigations of the whole complement of relevant biological molecules.
	
	Better understanding of phenotype-genotype relations.
	
	May provide insights into the impact of interactions between environmental conditions and genotypes, and mechanistic insights into disease aetiology.
	

**Limitations**	Limitations arising from cost of assays, quality of biological material available (e.g. instability of RNAs), and the amount of labour needed.
	
	Techniques still in their discovery state and new leads need to be carefully investigated and compared to existing biological information from in vivo and in vitro tests.
	
	New leads in the discovery of novel intermediate markers need to be confirmed in other independent studies preferably using different platforms.
	
	It is important to bear in mind that moving from promising techniques to successful application of biomarkers in occupational and environmental medicine requires not only the standardization and validation of techniques, but also appropriate study designs and sophisticated statistical analyses for interpretation of study results (i.e. issue of multiple comparisons and false positives).

There is also a challenge for accurate exposure assessment of environmental exposures with "-omics." Wild, for example, has expressed "the need for developing methods with the same precision for an individual's exposure, as we have for the individual's genome" [54]. He goes on to describe the concept of an 'exposome' which encompasses lifetime environmental exposure starting from prenatal period, and stresses the importance of even partial, targeted understanding of exposure, in addition to the genome, that could provide valuable advances. A prospective cohort study design can be carried out to best address the exposure biomarker approach, because it gives opportunities for repeat measures, as well as collection of samples before the onset of disease.

"-Omics" has the potential to play an important role in the generation of new insights into the etiology of disease and gene-environment interactions, but it is important to learn "what technologies can be usefully applied to which questions." [45] With the development of validated technologies, appropriate study designs, adequate sample size, inclusion of quality control and advanced statistical methods for data interpretation, "-omics" could potentially contribute significantly to the identification of environmental causes of disease and will help the field progress towards an integrated view of the interaction between environment and human health.

## List of abbreviations

GWAS: genome-wide association studies; ETS: environmental tobacco smoke; IARC: International; Agency for Research on Cancer; CVD: cardiovascular disease; NNK: 4-(methylnitrosamino)-1-(3-pyridyl)-1-butanone (NNK); POP: Persistent Organic Pollutant; RF-EMF: Radio-frequency modulated electromagnetic fields; INTERMAP: International study of macro- and micro-nutrients and blood pressure.

## Competing interests

The authors declare that they have no competing interests.

## Authors' contributions

PV and RV have drafted the manuscript, revised it critically and gave final approval of the version to be submitted.

JV has revised the manuscript and contributed to writing parts of it.

AK has contributed in writing the sections on "-omics", and also on revising the document.

All the authors have read and approved the final manuscript.
